# Considerations regarding a meta-analysis of laparoscopic total gastrectomy vs. laparoscopic-assisted total gastrectomy for gastric cancer

**DOI:** 10.1097/JS9.0000000000001025

**Published:** 2024-01-04

**Authors:** Miao Liu, Ai Shen, Huayang Pang

**Affiliations:** a Department of Gastrointestinal Cancer Center; b Chongqing Key Laboratory of Translational Research for Cancer Metastasis and Individualized Treatment; c Department of Hepatobiliary Pancreatic Tumor Center, Chongqing University Cancer Hospital, Chongqing, People’s Republic of China

*Dear Editor,*

We read with great interest the recent study titled ‘Safety and effectiveness of totally laparoscopic total gastrectomy vs laparoscopic-assisted total gastrectomy: A meta-analysis^1^’ published in *International Journal of Surgery*. This meta-analysis, comprising 15 studies with 3023 participants, demonstrated that totally laparoscopic total gastrectomy (TLTG) was significantly associated with reduced estimated blood loss, increased number of harvested lymph nodes and reduced hospitalization duration, compared to laparoscopic-assisted total gastrectomy (LATG), without compromising postoperative complications. The authors should be commended for providing the latest evidence on the feasibility of TLTG in gastric cancer. However, there are several concerns that require further clarification.

Firstly, the incidence of gastric cancer in China is among the highest globally, with a mortality rate accounting for about 40% worldwide. Chinese scholars have extensively explored and reported on the applicability of TLTG in gastric cancer, leveraging the objective advantage of large sample sizes. In this meta-analysis, it was noteworthy that a majority (7/15) of the studies were also published by Chinese researchers. However, we observed that the authors only included English articles while excluding four studies published in the Chinese language, potentially introducing significant publication bias artificially. Considering the availability of Chinese databases such as CNKI (China National Knowledge Infrastructure), Wan Fang (Wanfang Data), and VIP (Chongqing VIP Information Co., Ltd.) to the authors, it is recommended and encouraged to include Chinese studies after a thorough literature quality assessment.

Secondly, it was observed that the studies conducted by Zhong *et al*.^2^ (reference 11) and Zhao *et al*.^3^ (reference 12) were excluded by the authors due to their lack of exclusive focus on total gastrectomy. However, upon meticulous examination of the full texts and supplementary files of both articles, we found that they indeed performed subgroup analysis specifically pertaining to total gastrectomy and presented corresponding data. Consequently, it is imperative to incorporate these articles into the current pooled analyses.

Thirdly, among the studies included in this meta-analysis, we identified that both Lin *et al*.^4^ and Huang *et al*.^5^’s studies originated from the same medical center, with a significant overlap in their study periods. Consequently, incorporating these two studies simultaneously does not adhere to the principles of meta-analysis.

Finally, ensuring a good comparability of baseline characteristics is an essential prerequisite for drawing reliable conclusions. Regrettably, the authors did not provide detailed pooled analyses of the baseline variables between the TLTG and LATG groups encompassed in this pooled study. Therefore, whether the baseline parameters were adequately balanced across both cohorts remained unclear. Although subgroup analyses were conducted by the authors for types of stapler and neoadjuvant therapy, other factors such as age, BMI, and tumor size were not thoroughly examined regarding their significant impact on perioperative outcomes. This omission of these important variables may partially account for the authors’ inability to identify the sources of heterogeneity within the highly heterogeneous outcomes through meta-regression analyses.

## Ethical approval

No need for this letter to the editor.

## Sources of funding

This study was supported by the central university basic scientific research business expenses (2022CDJYGRH-008) and the Chongqing Science and health joint medical research project (2023MSXM104).

## Author contribution

M.L. and A.S.: original draft conception and writing; H.P.: critical revision of the manuscript. All authors reviewed the manuscript.

## Conflicts of interest disclosure

The authors have no conflicts of interest to declare.

## Research registration unique identifying number (UIN)

1. Name of the registry: none.
2. Unique identifying number or registration ID: none.
3. Hyperlink to your specific registration (must be publicly accessible and will be checked): none.

## Guarantor

Huayang Pang.

## Provenance and peer review

Commentary, internally reviewed.

## Data availability statement

Not applicable.
